# Domestic Saudi Arabian Travellers’ Understanding about COVID-19 and Its Vaccination

**DOI:** 10.3390/vaccines9080895

**Published:** 2021-08-12

**Authors:** Najim Z. Alshahrani, Sultan M. Alshahrani, Shehata Farag, Harunor Rashid

**Affiliations:** 1 Department of Family and Community Medicine, Faculty of Medicine, University of Jeddah, Jeddah 21589, Saudi Arabia; 2 College of Pharmacy, King Khalid University, Abha 61421, Saudi Arabia; shahrani@kku.edu.sa; 3 Department of Family and Community Medicine, King Khalid University, Abha 62529, Saudi Arabia; shehatafarag@yahoo.com; 4 Biostatistics Department, High Institute of Public Health, Alexandria University, Alexandria 21526, Egypt; 5 National Centre for Immunisation Research and Surveillance (NCIRS), Westmead, NSW 2145, Australia; harunor.rashid@health.nsw.gov.au; 6 Discipline of Child and Adolescent Health, The Children’s Hospital at Westmead Clinical School, Westmead, NSW 2145, Australia; 7 Marie Bashir Institute for Infectious Diseases and Biosecurity, School of Biological Sciences and Sydney Medical School, The University of Sydney, Westmead, NSW 2145, Australia

**Keywords:** airlines, COVID-19, preventive measures, travellers, vaccination, vaccine hesitancy

## Abstract

Background: There is a lack of data on Saudi domestic air travellers’ understanding regarding COVID-19 and their attitude towards the COVID-19 vaccination. Objectives: This study aimed to assess Saudi domestic air travellers’ understanding regarding COVID-19 and attitude towards mandating the COVID-19 vaccination for travellers. Methods: A survey using a self-administered, structured, and closed-ended questionnaire was conducted among domestic air travellers in Saudi Arabia. Participants’ socio-demographic information, travel history, health status, and attitudes and willingness to accept the COVID-19 vaccination were collected and analysed. Results: Of the 2236 respondents who participated in the survey, 542 (24.25%) had a history of COVID-19, 803 (35.9%) were exposed to a COVID-19 case, 1425 (63.7%) were concerned about catching COVID-19 during air travel, 796 (35.6%) thought the COVID-19 vaccination should be obligatory for travellers, 1105 (49.4%) thought it should be optional, and 335 (15.0%) thought the vaccination was unnecessary. Being of the male gender (adjusted odds ratio [aOR] 1.41, 95% confidence interval [95% CI] 1.14–1.69), being concerned about contracting COVID-19 (aOR 1.34, 95% CI 1.12–2.10) and frequent travelling (aOR 1.40, 95% CI 1.10–3.40) were predictors of vaccination uptake. Conclusion: This study demonstrates that although domestic Saudi travellers were concerned about COVID-19 infection, vaccine hesitancy was prevalent among them.

## 1. Introduction

COVID-19 is caused by severe acute respiratory syndrome coronavirus 2 (SARS-CoV-2) and may have a varied presentation ranging from asymptomatic infection to severe respiratory failure, pneumonia, and death [1,2]. The World Health Organization (WHO) declared COVID-19 a global pandemic on 11 March 2020 [3]; ever since, massive efforts to develop vaccines have resulted in several efficacious vaccines that are currently being rolled out across the globe. However, vaccine hesitancy is a great concern even in highly developed countries, jeopardising the global pandemic mitigation efforts [2,4]. A US survey suggests about 14.8% of respondents are unlikely to get vaccinated, and another 23.0% are unsure [5], making the control of the pandemic highly challenging; hence, public confidence is deemed an important determinant of the success of a vaccination campaign [6].

The COVID-19 pandemic has tragic consequences in terms of the death toll and economic and social chaos worldwide [7,8]. Since the start of the COVID-19 pandemic, the airline industry has been complying with strict sanitary requirements that resulted in numerous cancellations of flights, ensuring physical distancing and personal protective equipment for passengers and crews, and regular disinfection of carriers. Except for a few, most of the world’s borders have remained closed [9,10]. The Airports Council International (ACI) has estimated that in 2020, COVID-19 pandemic may have reduced passenger numbers by two-fifths and airport revenues by half. The International Civil Aviation Organization (ICAO) estimates that the gross loss of the airline industry is likely to be about US $112–135 billion. Additionally, the International Air Transport Association (IATA) expected very slow growth in the aviation industry in the second half of 2020, with an overall 55% reduction in passenger revenues [11]. Despite adherence of the airlines of the Gulf Cooperation Council (GCC) nations to COVID-19 protective measures, the need for travellers to be vaccinated is increasingly felt [12].

In Saudi Arabia, preventive measures against COVID-19, including social distancing and quarantine, are currently in place. Vaccination is considered the most effective strategy for limiting the transmission of COVID-19 to nurture positive health and, consequently, economic growth of a country [13]. A nationwide vaccination campaign has been launched in Saudi Arabia since December 2020 by the Ministry of Health. The country has a comprehensive preventive healthcare plan to offer domestic citizens and non-citizen residents free testing, treatment, and prevention of COVID-19. A mobile app called ‘Sehaty’ is being used to enroll in vaccination; vaccination hubs have been opened and in operation for vaccination across the country [14]. To curb an epidemic, achieving critical vaccination coverage is important [15]. However, according to the studies conducted in Saudi Arabia and elsewhere, the critical vaccination threshold is challenged by public hesitancy or unwillingness to accept vaccination [16,17]. Therefore, it is important to assess travellers’ understanding of COVID-19 and their willingness to accept the COVID-19 vaccination to formulate action plans to prevent the mass transmission of COVID-19 [18]. The current study aimed to assess domestic Saudi Arabian travellers’ understanding regarding COVID-19 and evaluate their attitude concerning mandating the COVID-19 vaccination for travellers.

## 2. Methodology

### 2.1. Study Design

This was a cross-sectional online survey conducted in the waiting areas of airports among Saudi Arabian travellers aged ≥18 years old who were travelling to domestic destinations by air. A survey questionnaire was developed following a literature review by searching for terms like ‘COVID-19 vaccination’, ‘survey,’ ‘attitude,’ ‘understanding’ in Medline, and an expert consultation using the Google template panel. Volunteer medical students (data collectors) were recruited to distribute the survey link using a barcode among travellers in six domestic airports in Saudi Arabia, namely Riyadh, Dammam, Jeddah, Abha, Tabuk, and Bisha, from 25 February to 15 March 2021. Due to COVID-19 related restrictions, a convenience sample technique was utilised for this study. These restrictions included: limitation on exceeding a certain number of people in a specified room/floor, minimising the number of onsite staff in the airports; limited access to food court areas, and measures to reduce hand contact methods; therefore, barcodes linked to the questionnaire were hung in the waiting areas of the airport, and the participants were asked to fill out the questionnaire. The data collectors explained the study to the adult travellers who could potentially participate in the study. Before beginning the distribution of the survey questionnaires, the data collectors explained the study to the potential participants, who were then requested to complete an online informed consent template. The survey took on average about five minutes to complete. A formal sample size calculation was not deemed necessary for this online survey; in similar Saudi Arabian studies, a sample size of about 2000 participants was a representative sample [19,20].

### 2.2. Measures

The questionnaire was self-administered, structured, and contained closed-ended questions. It was devised following the examples of several previous studies that dealt with the COVID-19 vaccination [19,20,21,22,23,24]. The questionnaire was then adjusted to suit Saudi Arabian travellers and was pilot tested to ensure its validity. A language expert was consulted to ensure the accuracy of the survey questions. In order to make data collection more convenient, the survey was also translated into Arabic. The questionnaire had four parts. The first part summarised the study goals, outcomes, and what was involved in participating in the survey, and it concluded with an informed consent declaration to proceed. The second part included the participants’ socio-demographic information as well as their past travel history. The third part had questions about participants’ exposure to COVID-19, current travel information, health status, and past medical history. In this section, participants were asked about the airlines they were travelling with, their attitude to COVID-19 vaccination, and how it could impact their travel plans. In the fourth part, participants were asked if they would like to see any preliminary findings from the report, and finally, they were thanked for their cooperation.

### 2.3. Data Analysis

The data from the questionnaires were checked for accuracy, consistency, and completeness before being cleaned, coded, and entered into SPSS version 20 (IBM Corp., Armonk, NY, USA). Descriptive analysis was used to present the categorical variables (e.g., socio-demographic factors) as frequencies and percentages. Continuous variables (e.g., age) were presented as means with standard deviation (SD). Multiple logistic regression analysis was carried out to identify determinants of intention to receive the COVID-19 vaccination for travel.

## 3. Results

### 3.1. Participant Characteristics

Of the 2321 adult travellers who responded to the study, 2236 completed the survey (96% completion rate). The respondents were aged 18 to 65 years (mean 26.2 ± SD 12.4 years), 1220 (54.6%) were female, 1283 (57.4%) were married, 1606 (71.8%) had completed university-level education, 1001 (44.8%) were employed and 521 (33.4%) were health care workers. Of all, 1125 (50.4%) participants had a monthly income of ≥5000 Saudi Riyals (equivalent to US $1740) (Table 1).

### 3.2. COVID-19 Infection and Prior Air Travel

Out of all respondents, 542 (24.2%) participants had COVID-19, 803 (35.9%) reported contact with a COVID-19 case, 1812 (81%) had travelled by air at least once in the year before the pandemic, 1698 (79.9%) were concerned about contracting COVID-19 during travel, and 1951 (87.3%) reported COVID-19 pandemic affected their travel plans (Table 2).

### 3.3. Attitude towards Compliance with Preventive Measures

Table 3 summarises participants’ attitudes towards air travel and COVID-19 vaccination stratified by their travel frequency. Most travellers (64.3% of frequent travellers and 69.4% of infrequent travellers) believed airlines were complying with necessary precautions to prevent the spread of COVID-19. Approximately half of the respondents thought that COVID-19 vaccinations should be prioritised for travellers, 35.6% respondents thought the COVID-19 vaccination should be obligatory for travellers, 51.4% respondents would travel by plane despite not being vaccinated, 73.1% travellers thought medical insurance must cover vaccination against COVID-19 and its treatment, and 40.2% travellers thought air travel should be minimised despite mass vaccination.

In multiple logistic regression, male gender (adjusted odds ratio [aOR] 1.41, 95% confidence interval [95% CI] 1.14–1.69), being concerned about contracting COVID-19 during travel (aOR 1.34, 95% CI 1.12–2.10), and frequent travelling (aOR 1.40, 95% CI 1.10–3.40) were associated with a significant likelihood of having the intention to receive the COVID-19 vaccination for travel. Meanwhile, being older (aOR 0.89, 95% CI 0.80–0.99) and being married (aOR 0.75, 95% CI: 0.60–0.94) were associated with a significant likelihood of having no intention to be vaccinated. Education (aOR 1.11, 95% CI: 0.93–1.33), employment (aOR 0.95, 95% CI: 0.76–1.19), and income (aOR 1.06 95% CI: 0.92–1.22) were not related to intention to receive COVID-19 vaccine (Table 4).

## 4. Discussion

This study shows that a large proportion of survey participants used airlines (81% at least once a year) for travel, about a quarter had a history of suffering from COVID-19, over a third had a history of exposure to a COVID-19 case, indicating a potential risk of travel-related COVID-19 transmission among domestic air passengers without appropriate preventive measures. However, some ‘noncompliant passengers’ were ready to travel despite not complying with precautionary measures against COVID-19: for instance, about half of the respondents admitted that they travelled despite not receiving the vaccination, about one in nine passengers thought their decision to travel would not be affected even if the airlines did not comply with preventive measures, and about 5% passengers did not have any preference for airlines with better compliance with precautionary measures. However, this group was a small minority. Over three-quarters of the travellers were concerned (64% were greatly concerned) about contracting COVID-19 during travel, and over half agreed that the COVID-19 vaccination should be prioritised for travellers. Two-thirds thought travel insurance was important for air passengers, and 73% said such insurance should cover COVID-19 vaccination and treatment for COVID-19. In addition, most participants admitted that their travel plans were affected by the pandemic. Predictors of uptake of vaccination included being male and concerned about contracting COVID-19 during travel and being a frequent traveller. Misperception existed among travellers, with 49% reporting disbelief or concern surrounding COVID-19 vaccination, and about 65% considering the vaccination not mandatory.

The risk of air travel-related COVID-19 transmission should not be underestimated, as ‘mass transmission events’ (defined as >1 secondary case) have been reported during air travel [18,25,26]. During this COVID-19 pandemic, global authorities put restrictions on air travel to limit the transmission of the virus. Infection control measures are implemented by most airlines [27], but currently, there is a large push from trade, commerce, tourism, and other related sectors to lift the air travel ban [28]. By the end of 2020, the airline closures led to about 1.7% global gross domestic product (GDP) loss, with about 25 to 30 million job cuts. Leading tourist countries have implemented a risk-based strategy to reopen airline industries. Still, a high prevalence (up to 6.3%) of asymptomatic SARS-CoV-2 infection among passengers of repatriation flights from Europe indicates an occurrence of a silent air travel-related COVID-19 transmission [28]. A quarter of our cohort had COVID-19, and a third had a history of exposure to COVID-19 patients indicating a likely risk of travel-related COVID-19 transmission among Saudi Arabian domestic air travellers.

Although half of the participants in this survey reported travelling for tourism or entertainment, about one in nine ‘noncompliant passengers’ thought their decision to travel would not be influenced by airlines’ adherence or non-adherence to preventive measures. Another 5% of passengers were not worried by airlines’ better adoption of precautionary measures than other airlines. That a large proportion of university-educated individuals who were unemployed travelled for entertainment or tourism sounds at the outset counterintuitive. Still, this survey was conducted during school holidays when people tend to go on a vacation. In Saudi Arabia, most unemployed fresh graduates get financial assistance from the government for up to two years. It is likely that many such individuals took a holiday within the country immediately after opening the domestic flights after almost a year of closure (when international flights were still closed). About half of the respondents admitted that they would travel despite not receiving the vaccination. Almost half considered the COVID-19 vaccination “optional for travellers,” 15% thought vaccination was unnecessary, which may have stemmed from overall vaccine hesitancy in Saudi Arabia. Several vaccines are being rolled out worldwide, and the Saudi Arabian Ministry of Health has approved the Pfizer-BioNTech vaccine (Comirnaty) since mid-December 2020 [14]. A social media app (called Tawaklna) is being used to raise public awareness. A self-administered survey conducted in four Arab countries, including Saudi Arabia, showed about 33% of participants were hesitant to receive the COVID-19 vaccine. An electronic survey of health care workers in Saudi Arabia showed only 61.2% were willing to receive the COVID-19 vaccination; vaccine safety was the strongest predictor of willingness to receive the COVID-19 vaccination [29]. A cross-sectional survey involving 1333 Saudi Arabian respondents showed about 72% would receive the COVID-19 vaccination, whereas another survey involving 2137 respondents showed only 48% were willing to receive it [17]. Finally, a web-based survey involving 992 Saudi Arabian respondents showed 65% were interested in accepting the COVID-19 vaccination [19]; in reality, as of early February 2021, the actual vaccination rate has been only 2.1%, with another 20.4% booked for vaccination [14].

This study also showed that being concerned about being at risk of COVID-19 during travel (aOR 1.34 [1.12–2.10]) was a significant predictor of COVID-19 vaccination, which is bolstered by another study that showed perceived susceptibility was an important predictor of uptake of vaccination (OR 3.82 [1.64–8.94]). The male gender was also associated with a higher likelihood of receiving the vaccination because of higher susceptibility and severity risk among males [30]. Another reason for greater uptake among males was that they tend to be more active in outdoor activities, and travel more often than females in Saudi Arabia’s conservative culture. In other surveys, compared to males, more females were unsure about the safety of the COVID-19 vaccination, and fewer females compared to males had access to information resources [31,32].

WHO has recently issued guidelines on resuming international travel [33]. Vaccination against COVID-19 is now considered mandatory for international travel [34,35]. At the same time, the use of certifying documents such as vaccination or immunity passports has also been suggested [27,36]. Hygienic methods, mask use, and physical distancing are also considered effective measures during air travel [37]. An expert assessment report prepared by the Faculty and Scientists at the Harvard T.H. Chan School of Public Health state a layered approach comprised of continuous operation of ventilation systems, surface disinfection, universal wearing of face masks by passengers and crews, and implementation of procedures during boarding and deplaning to maximise social distancing could reduce the risk of SARS-CoV-2 transmission in airplanes [37,38]. Despite the benefits of those hygienic measures, to approximately 13% of respondents, whether an airline adheres to precautionary measures would not matter. To about 5% of respondents, whether an airline performs better or worse in complying with preventive measures would not matter.

Over two-thirds of the participants agreed that health insurance is required for airline travellers, and about 73% of respondents thought such insurance should cover COVID-19 vaccination and treatment. However, many insurers have already ceased offering travel insurance since the pandemic, although some have since resumed, many insurers are still refusing to cover COVID-19 treatment and prevention [39].

The study’s strength is that it is the first study of its kind to assess domestic Saudi travellers’ understanding of COVID-19 and the vaccinations available. However, it has some limitations. Due to the ongoing pandemic, a convenience sampling strategy was used, which cannot be replicated and is nongeneralisable. Moreover, the information collected was based on anecdotal data and was not verified; hence, the results need cautious interpretation. Finally, the survey design was such that the respondents were free to skip a question if they did not want to answer. This resulted in an incomplete response to some questions (e.g., reason for travel), which may have led to some attrition bias.

Future studies should focus on exploring obstacles, attitudes, and challenges to COVID-19 vaccination among domestic Saudi Arabian travellers.

## 5. Conclusions

The study provides an insight into Saudi Arabian domestic travellers’ attitudes towards COVID-19 vaccination requirements for travel. It demonstrates that the COVID-19 pandemic affects their travel plans. Although most respondents favour prioritising COVID-19 vaccination for travellers, the majority do not favour mandating the COVID-19 vaccine for travel, and misperception around the vaccine prevails. Vaccine hesitancy among Saudi travellers should be addressed to ensure optimum COVID-19 vaccine uptake.

## Figures and Tables

**Table 1:** Socio-demographic information of the survey respondents (N = 2236).

Socio-Demographic Data	Number (%)
Age in years	
18–25	958 (42.8)
26–35	667 (29.8)
36–45	360 (16.1)
46–54	176 (7.9)
*55+*	75 (3.4)
Gender	
Male	1016 (45.4)
Female	1220 (54.6)
Marital status	
Single	953 (42.6)
Married	1283 (57.4)
Education	
Below secondary	87 (3.9)
Secondary	543 (24.3)
University/above	1606 (71.8)
Employment	
Not currently employed	1235 (55.2)
Currently employed	1001 (44.8)
Occupation	
Non-health care sector	1037 (66.6)
Health care sector	521 (33.4)
Nature of occupation	
Sedentary work with no contact with clients	253 (23.5)
Sedentary work with direct contact with clients	530 (49.2)
Outdoor work	295 (27.4)
Monthly income	
<5000 SR	1111 (49.7)
5000–10,000 SR	567 (25.4)
>10,000 SR	558 (25.0)

SR = Saudi Riyal.

**Table 2:** Participants’ history of COVID-19 infection and past air travel.

COVID-19 Exposure and Past Travel	Number (%)
Infected with COVID-19 (n = 2236)	
Yes	542 (24.2)
No	1694 (75.8)
Had contact with COVID-19 case but did not develop infection (n = 2236)	
Yes	803 (35.9)
No	1433 (64.1)
Before the COVID-19 pandemic, how often did you travel by air? (n = 2236)	
Never	424 (19.0)
1 time/year	1045 (46.7)
2–3 times/year	525 (23.5)
>3 times/year	242 (10.8)
What is the main reason for you to travel by air? (n = 1812)	
Business/trade	158 (7.1)
Tourism	1117 (50.0)
Study	267 (11.9)
Others	270 (12.1)
How concerned are you about contracting COVID-19 infection during air travel? (n = 2236)	
Not concerned at all	538 (24.1)
To some extent concerned	1425 (63.7)
Highly concerned	273 (12.2)
Do you have any disbelief in or concern about the COVID-19 vaccination? (n = 2236)	
Yes	1096 (49.0)
No	1140 (51.0)
Has COVID-19 pandemic affected your travel plans? (n = 2236)	
No effect at all	285 (12.7)
Some effect	1044 (46.7)
Significant effect	907 (40.6)

**Table 3:** Distribution of participant’s attitudes towards air travel and COVID-19 vaccine by their annual travel frequency.

Attitude towards Air Travel and Vaccination		Total	Annual Travel Frequency		*p* Value
			Infrequent	Frequent	
		N (%)	n (%)	n (%)	
I believe airlines are taking appropriate precautions to prevent the spread of COVID-19 safely and effectively.	Disagree	117 (5.2)	62 (4.2)	55 (7.2)	0.004 *
	Neutral	606 (27.1)	387 (26.3)	219 (28.6)	
	Agree	1513 (67.7)	1020 (69.4)	493 (64.3)	
I think travellers should be prioritised for COVID-19 vaccination.	Disagree	379 (16.9)	228 (15.5)	151 (19.7)	0.006 *
	Neutral	685 (30.6)	438 (29.8)	247 (32.2)	
	Agree	1172 (52.4)	803 (54.7)	369 (48.1)	
I decided to travel by plane even if I did not get COVID-19 vaccine	Disagree	545 (24.4)	399 (27.2)	146 (19.0)	0.001 *
	Neutral	542 (24.2)	370 (25.2)	172 (22.4)	
	Agree	1149 (51.4)	700 (47.7)	449 (58.5)	
My travel plans have changed after the announcement of COVID-19 vaccination rollout	Disagree	386 (17.3)	255 (17.4)	131 (17.1)	0.641
	Neutral	804 (36.0)	537 (36.6)	267 (34.8)	
	Agree	1046 (46.8)	677 (46.1)	369 (48.1)	
What do you think about mandating the COVID-19 vaccination for air travellers?	No need as existing measures are sufficient.	335 (15.0)	207 (14.1)	128 (16.7)	0.035 *
	Should be optional.	1105 (49.4)	713 (48.5)	392 (51.1)	
	Should be obligatory.	796 (35.6)	549 (37.4)	247 (32.2)	
My decision to travel by an airline will be affected by the extent to which it adheres to precautionary measures irrespective of mandatory vaccination	Disagree	294 (13.1)	168 (11.4)	126 (16.4)	0.004 *
	Neutral	616 (27.5)	416 (28.3)	200 (26.1)	
	Agree	1326 (59.3)	885 (60.2)	441 (57.5)	
Medical insurance is important for air travellers	Disagree	200 (8.9)	117 (8.0)	83 (10.8)	0.077
	Neutral	526 (23.5)	352 (24.0)	174 (22.7)	
	Agree	1510 (67.5)	1000 (68.1)	510 (66.5)	
Air travellers’ medical insurance must cover obtaining the COVID-19 vaccine or treatment in the event of infection during the course of travel	Disagree	158 (7.1)	90 (6.1)	68 (8.9)	0.024 *
	Neutral	444 (19.9)	283 (19.3)	161 (21.0)	
	Agree	1634 (73.1)	1096 (74.6)	538 (70.1)	
The decision to travel by a particular airline is affected by how it adheres to precautionary measures compared to other airlines	Disagree	111 (5.0)	69 (4.7)	42 (5.5)	0.437
	Neutral	555 (24.8)	356 (24.2)	199 (25.9)	
	Agree	1570 (70.2)	1044 (71.1)	526 (68.6)	
I think air travel should be minimised in near future despite widespread vaccination	Disagree	753 (33.7)	449 (30.6)	304 (39.6)	0.001 *
	Neutral	585 (26.2)	381 (25.9)	204 (26.6)	
	Agree	898 (40.2)	639 (43.5)	259 (33.8)	

* *p* < 0.05 (significant).

**Table 4:** Multiple logistic regression models for determinants of intention (obligatory) to have the COVID-19 vaccine on travel.

Factors	aOR	95% CI	
Age in years	0.89	0.80	0.99
Male gender	1.41	1.14	1.69
Married	0.75	0.60	0.94
Education	1.11	0.93	1.33
Working	0.95	0.76	1.19
Income	1.06	0.92	1.22
Concerned about catching the COVID-19 infection or being in contact with an infected case during airline travel	1.66	1.42	1.94
Not infected with COVID-19	1.34	1.12	2.10
Had contact with a COVID-19 case but did not develop infection	1.10	0.90	1.20
Frequent travelling	1.40	1.10	3.40
Has the spread of the new coronavirus affected your travel plans?	1.20	1.10	1.50

aOR = adjusted odds ratio.

## Data Availability

Data available upon request.
